# Sneezes and Stitches

**Published:** 1893-05

**Authors:** 


### SNEEZES AND STITCHES.

It may be well to know that the very considerable inconvenience, *a stitch in the side*, is often removed in the same manner that a sneeze is prevented, to wit, by expiring all the breath possible from the lungs; push out the breath for half a minute or more, and draw it in slowly.

